# The view of Hong Kong parents on secondary use of dried blood spots in newborn screening program

**DOI:** 10.1186/s12910-022-00839-z

**Published:** 2022-11-01

**Authors:** L. L. Hui, E. A.S. Nelson, H. B. Deng, T. Y. Leung, C. H. Ho, J. S.C. Chong, G. P.G. Fung, J. Hui, H. S. Lam

**Affiliations:** 1 Department of Applied Biology and Chemical Technology, The Hong Kong Polytechnic University, Hung Hom, Hong Kong SAR, China; 2 Department of Paediatrics, Faculty of Medicine, The Chinese University of Hong Kong, Shatin, Hong Kong SAR, China; 3 Department of Obstetrics and Gynaecology, Faculty of Medicine, The Chinese University of Hong Kong, Shatin, Hong Kong SAR, China; 4 Academia Sinica, Taipei, Taiwan; 5 Private paediatrician, Hong Kong SAR, PR China

**Keywords:** Newborn screening, Residual dried blood spots, Biobanking, Parental autonomy, Informed consent, Data privacy

## Abstract

**Background:**

Residual dried blood spots (rDBS) from newborn screening programmes represent a valuable resource for medical research, from basic sciences, through clinical to public health. In Hong Kong, there is no legislation for biobanking. Parents’ view on the retention and use of residual newborn blood samples could be cultural-specific and is important to consider for biobanking of rDBS.

**Objective:**

To study the views and concerns on long-term storage and secondary use of rDBS from newborn screening programmes among Hong Kong Chinese parents.

**Methods:**

A mixed-method approach was used to study the views and concerns on long-term storage and secondary use of rDBS from newborn screening programmes among Hong Kong Chinese parents of children 0–3 years or expecting parents through focus groups (8 groups; 33 participants) and a survey (n = 1012, 85% mothers) designed with insights obtained from the focus groups. We used framework analysis to summarise the themes as supportive factors, concerns and critical arguments for retention and secondary use of rDBS from focus group discussion. We used multiple logistic regression to assess factors associated with support for retention and secondary use of rDBS in the survey.

**Results:**

Both in focus groups and survey, majority of parents were not aware of the potential secondary use of rDBS. Overall secondary use of rDBS in medical research was well accepted by a large proportion of Hong Kong parents, even if all potential future research could not be specified in a broad consent. However parents were concerned about potential risks of biobanking rDBS including leaking of data and mis-use of genetic information. Parents wanted to be asked for permission before rDBS are stored and mainly did not accept an “opt-out” approach. The survey showed that parents born in mainland China, compared to Hong Kong born parents, had lower awareness of newborn screening but higher support in biobanking rDBS. Higher education was associated with support in rDBS biobanking only among fathers.

**Conclusion:**

Long-term storage and secondary use of rDBS from newborn screening for biomedical research and a broad consent for biobanking of rDBS are generally acceptable to Hong Kong parents given their autonomy is respected and their privacy is protected, highlighting the importance of an accountable governance and a transparent access policy for rDBS biobanks.

## Background

### Newborn screening for inborn errors in metabolism using dried blood spots (DBS)

Newborn screening using DBS aims to detect pre-symptomatic inborn errors in metabolism so that early therapeutic interventions can be implemented to prevent severe illness or premature death. It usually takes place within 24–48 h of a child’s birth and it involves placing a few drops of blood obtained from a heel prick on special pre-printed filter paper that preserve the blood sample as dried blood spots (DBS). The few drops of blood are usually more than enough for the screening purpose, and the remaining DBS are called residual dried blood spots (rDBS).

### Secondary use of DBS

With the growth of newborn screening programmes and advances in omics technology, rDBS represent a valuable, powerful and cheap resource for medical, clinical and public health research. rDBS are an optimal resource for genetic and epigenetic studies, as DNA can be extracted from DBS even after long-term storage. In settings where uptake of newborn screening is high, rDBS offers an unbiased population sample suitable for population-based research such as prevalence studies. There has been effort in turning rDBS collection into a biobank (e.g. Danish newborn screening biobank) [1] The scientific community sees such large scale biobanks as having great scientific potential for understanding health and disease.[2].

### A consent challenge

The use of rDBS from newborn screening offers opportunities as well as challenges.(3) Firstly, the rDBS contains DNA, thus their use potentially threatens individual privacy by revealing health and genetic information about an individual.(4) Secondly, there has been debate over types of consent from parents for the use of rDBS outside screening purposes. A broad initial consent accompanied with appropriate governance has been an emerging model within the population biobank community. However it may not be universally accepted.(5) Given the ethical ambiguities associated with the retention and use of residual newborn blood samples,(6) some controversies related to consent have even escalated to lawsuits that jeopardise the trust of the parents in newborn screening itself.(7) These issues emphasise the need to address cultural-specific public opinion and concerns, particularly from parents,(2) in the process for developing policies for newborn screening programmes and secondary use of rDBS.(8)

### Views of Hong Kong parents

In Hong Kong, there is no legislation for biobanking. An expanded newborn screening has been implemented in Hong Kong over the past few years and has become part of the routine care for all newborns delivered in public hospitals at no cost from 2021.(9, 10) In Hong Kong, all parents are given information about newborn screening during the antenatal period and they give informed consent to let their newborns take part after childbirth. Policies for storage of rDBS are different by public and private birthing hospitals. rDBS from the expanded newborn screening for children born in public hospitals are stored for 2 years for newborn screening related purposes after which they would be destroyed. On the other hand, the expanded newborn screening for children born in private hospitals is provided by a private laboratory which seeks parents’ consent to store the rDBS for longer for medical research after removing all identifying information. Here we studied the views of Hong Kong parents about the secondary use of rDBS to inform policy framework that facilitates secondary use of rDBS with consideration of cultural-specific ethical concerns in Hong Kong.

## Methods

We studied the views of parents about the retention and secondary uses of rDBS using a mixed-method approach, i.e. we collected qualitative data via focus group discussions and quantitative data via a survey. In this study, the term “secondary use of rDBS” was defined as “the use of rDBS for a purpose different from the purpose that it was originally collected for”.

### Study population

We recruited parents of young children (0–3 years old) or expecting parents, who were Chinese, 18 years old or above and able to read Chinese (and speak Cantonese for focus groups). The participants were either (1) mothers of newborns in the postnatal wards in two public hospitals, (2) parents of 0–2 years who took part in another project “Hong Kong Growth Study” and consented for further contact for other health-related research and (3) parents whose children aged 0–3 years attending kindergartens or child-care centres in Hong Kong.

### Focus groups

We conducted semi-structured focus group discussions via the Zoom video-conferencing platform. Standardised background information on newborn screening, including its purpose of newborn screening, the procedure of taking DBS and the potential use of rDBS as well as the questions to ask and ground rules for discussion was given to participants a week before the discussion.

The discussion was led by a research assistant using standard structured questions focusing on the opinions about length of retention, ownership of rDBS, types of secondary uses, risks and benefits of research using rDBS, ethical considerations, types of parental consent and governance, transparency, autonomy and confidentiality using simple stimulus materials. The discussion was mainly guided but the participants were free to discuss issues that naturally arose. We stopped recruitment when saturation of themes was reached. As a token of appreciation, each participant in the focus groups received a cash coupon of HKD200 (USD 25).

### Survey

A questionnaire was designed based on a review on similar studies in other countries [11–15] and findings from our focus group discussions. The questionnaire focused mainly on parents’ views about anonymous secondary use of rDBS including prevalence studies, test development, linkage to medical records, epidemiological studies, biobanking, DNA sequencing, anonymous research by third parties (e.g. insurance, pharmaceutical and biotechnology companies) and unknown future research. We also ask parents’ opinion on a few non-anonymous secondary uses i.e. identifying victims or tracing suspects of crimes. Other information including age, number of children, place of birth, education attainment, occupation, household income, religion were collected.

### Data analysis

***For Focus Groups -*** We transcribed the data and we adopted key steps suggested by the framework analysis methodology to analyse the data. [16] The transcripts were coded into predetermined themes including supportive factors, concerns and critical arguments for retention and secondary use of rDBS that previous studies suggested. Data that could not be coded into one of the predetermined themes was coded with new categories. The codes were linked together until all the data could be allocated into distinct themes. Initial coding was carried out by two research staff carrying out the focus group discussion. All codes and themes were compared for consistency and discrepancies were further discussed with the first author for consensus. The analysis was carried out with Microsoft Excel spreadsheets so as to allow cross tabulation of individual responses against identified themes [17].

***For the Survey -*** The parents’ view on and concerns of retention of rDBS samples, secondary use of rDBS and types of parental consent were reported. We used multivariable logistic regression to assess factors, including sex, age, place of birth (Hong Kong or the Mainland), education attainment, household income and religion, associated with support for retention and secondary use of rDBS. Statistical analyses were performed using R version 3.1.2 (R Development Core Team, Vienna, Austria).

### Ethics approval

The study was reviewed by and received approval from the Joint Chinese University of Hong Kong-New Territories East Cluster Clinical Research Ethics Committee <CREC Ref. No. 2019.492> and the Research Ethics Committee (Kowloon Central/Kowloon East) < KC/KE-20-0057/ER-4>. Written informed consent/e-consent were obtained from the participating parents. The study was conducted in accordance with the relevant guidelines and regulations, including Declaration of Helsinki.

## Results

### Focus groups

We conducted eight semi-structured focus groups via the Zoom platform in 2020. Each focus group involved 4–5 parents and each discussion session lasted for about 120 min. There were 33 participants in total, comprising 27 women and 6 men, with a mean age of 35 years. The majority (80%) of the participants were university graduates. Most of them were not aware that the rDBS from newborn screening could be stored for secondary use before reading the pre-discussion materials.

We summarised the opinions of participants, as (1) supportive factors (personal and family interests, altruism and trust in the governance body), (2) concerns (privacy and lack of trust in the governance body) and other arguments, including (3) autonomy and (4) transparency of policy related to retention and secondary use of rDBS.

### Supportive factors for retention and secondary use of rDBS

#### Personal and family interests

The support for storage and use of rDBS often links with the personal and family interests of the participants. Parents who support storing rDBS for personal interest also support storing the personal information with rDBS for further possible contact.

P014: “Genetic diseases can be inherited across generations. This will help to find out the origin of such genetic diseases.”

P030: “If you choose option 2 (anonymous storage), the storage will be less useful. For example, the family cannot be contacted even if there is a cure (for the illness discovered).”

P029: “This (anonymous storage) will not allow prevention of premature death in the next pregnancy.”

#### Support because of altruism

Some participants supported the retention and secondary use of rDBS because they perceived certain potential benefits of rDBS to others, i.e. a sense of altruism. In general there is a support for the secondary use of rDBS in medical research so as to improve public health.

P004: “I agree to use it for medical purposes. I want to give back to the society and help sick children in the future.”

P014:“I support permanent storage because this will help with developing drugs to treat new diseases and this will help children and other people suffer from such new diseases.”

Although the majority of the participants still highly valued their privacy and autonomy, the presence of altruism drove some participants to relax the personal control in the consenting process, e.g. supporting the use of opt-out approach and not supporting the re-consenting from adult child:

P030: “Sufficient information is needed for medical research, so I think all data should be stored. The rDBS will not be useful if some parents don’t have the knowledge (about what information is needed for research) and they object (to store all data).”

P030: “(I think) there is no need (to re-consent by adult children) as perhaps concern is little when this is for scientific research. The data will be much less after seeking re-consent.”

P014: “(If children do not indicate their wish when they become adults), I think the stored rDBS should continue to be kept by default. It is a real pleasure to help others and medical research.”

Similarly some participants showed altruism also showed more support for longer term storage and sharing of personal information:

P037: “Option 2 (anonymous storage) does not allow certain usage (of rDBS), such as tracing missing persons, and it is wasteful. Option 1 (identified storage) allows more flexibility in usage (of rDBS).”

P031: “(Permanent storage) makes the sample pool bigger and more useful.”

#### Trust in the governance body

We observed that trust in the users/governance body of rDBS influenced the participants’ support for secondary uses of rDBS. There was a greater trust in professional and academic institutions and their use of rDBS compared to the government and commercial companies.

P026: “For the time being I trust universities and other research institutions in Hong Kong. I trust the Chinese University of Hong Kong. ”

P016: “It is safer to have people with professional knowledge to store and utilise (rDBS), so it will not be messed up.”

P003: “It would be ideal to involve professional organizations (in the governance or rDBS). It is quite difficult for Hong Kong people to accept the government (being involved).”

P029: “I am not very comfortable if the government owns and manages this (rDBS bank). The huge current unrest in the society makes me worried. It would be better if there are different stakeholders involved.”

P031: “I don’t know if they (commercial companies) will use our data for biotechnology purposes, gene editing, or things that violates the law. In addition, I am unsure how much the new drug from pharmaceutical companies will benefit us, or whether the new drugs will only be affordable by the privileged and not for the benefits of the general public.”

Besides the type of users, the perceived resources and efficiency of the users also influence parents’ trust. For example, some believed that commercial organisations may work more efficiently with rDBS:

P012: “Is it possible that some research cannot be carried out by non-profit institutions due to a lack of funding and can be carried out by profit-making companies? If so I can accept it (commercial companies).”

### Concerns about retention and secondary use of rDBS

#### Privacy issues

Privacy of personal data and genetic information was the major concern of participants when considering biobanking rDBS for future use. Such concerns have implications on their preference on the types of secondary uses and storage methods that do not use personal data.

P043: “I choose option 2 (anonymous) due to privacy issues. I don’t know if my child is sick, so it is better to keep private.”

P041: “I do not support with option 7 (tracing crime suspects). This is a privacy issue. It seems that they take the information without informing us. Option 6 (identifying victims) is more acceptable (to me), and some families may want to use them.”

P042: “I feel that the crime investigation should be performed by relevant bodies, and they should not use privately stored blood for this purpose. And using (privately stored blood) without consent is like invading privacy.”

P037: “Although I agree (to use it for medical purposes), I only agree on anonymous research. I do not agree with non-anonymous research, as disclosing the identity may potentially affect the children, e.g. their insurance coverage, or being known to have AIDS. (Are you worried about discrimination?) Yes.”

#### Lack of trust in government

These concerns about privacy leakage are to some extent related to participants’ perception of the social and political environment in Hong Kong when the focus group was conducted. Some participants indicated that they perceived higher potential risks of sharing rDBS partly due to the recent social unrest and lack of trust in the police.

P030: “In the current political environment, we are worried that if rDBS are being used for option 7 (tracing crime suspects), they (the police) will be given the opportunities to do things we don’t want them to do.”

P008: “Now we don’t believe much in the government and government-related institutions. We don’t know what they are going to do with your information in the future.”

P029: “The (Hong Kong) society is currently (not stable) filled with turmoil. (We) may participate in some social movements, and someone drop your blood to wrong people (who took part in social movements). So I feel hesitate with option 7 (tracing crime suspects).”

Participants also thought that the involvement of parents in the governance process is important so that they can take more control in the governance.

P018: “I prefer to have more voices in it, rather than only having the government processing and manipulating the database. Whether parents should participate can be discussed later. I support more people to supervise this organization.”

P030: “The most important thing is to have parents, who provide samples, take part in it (the governance). There is no reason not having a means for the sample providers to make a voice.”

### Autonomy

Due to the concerns on data privacy and mistrust in the government, participants highly valued the autonomy of the donors of rDBS (both parents and children) in the discussion concerning consenting issues.

P037: “We should make this decision by ourselves, but not by others.”

P039: “I think if I can choose not to [have the sample] be stored, I feel I am more respected… (This is) autonomy.”

The majority preferred the opt-in approach (where rDBS will only be stored when parents consent) so they could have personal control and give clear permission before their children’s rDBS are stored. They did not think opt-out approach (where rDBS are stored for secondary use unless parents object) is acceptable.

P043: “When it comes to privacy, it’s better to ask for parents’ consent.”

P005: “I want to add that consent should be obtained from participants in advance (even) for medical use.”

P026: “I choose “Opt-in”. If I trust the society and the government, I will choose “Opt-out”.”

Some preferred multi-layer consent / dynamic consent and a mechanism to allow withdrawal of consent so as to have greater personal control.

P034: “I don’t want to give single consent for all, as it’s like a “buyout” to me. There should be some rules such that we can choose to withdraw later. With new technology, there may be some research and uses that we cannot foresee now.”

P043: “Maybe it is better to regulate the scope of uses, i.e. it is limited to certain uses in the beginning. And you will be notified when there are new uses.”

P009: “Yes, there should be a form to make changes (on the initial consent). There is a need for an option to revise (consent), although I believe not many people would bother to change.”

There was a general agreement that the biobanks should obtain a fresh consent from an adult child to respect their autonomy.

P029: “When he (the child) is an adult, he has the right to decide, and he should decide whether to store it or destroy it immediately.”

P017: “The rDBS belongs to my child and not me. I help him decide when he is under 18 years old. He may not want to do this when he grows up.”

### Transparency

Participants perceived risks on an unaccountable governance body and unauthorised use of rDBS. They iterated the importance of transparent regulations with specific research scope while discussing the governance process and the consent mechanism of such a biobank.

P037:“It is safer (if governance is described in the consent), otherwise, it seems that some genetic research without regulation i.e. those we learnt in the newspaper, may happen.”

P042: “The government seems to govern many things, but in reality there is little governance. If it (the governance) is described clearly, I could look into responsibility if needed.”

### Survey

A total of 861 mothers and 151 fathers (total 1012 parents with mean age of 33.8 years) took part in the survey, from whom about half (53%) had one child. The majority of the parents were born in Hong Kong (69%) or mainland China (29%). Our respondents were relatively higher educated compared to the 2016 population of similar age and sex, with 45% having a university degree or above. (Table 1) The majority (76%) of respondents were aware that their youngest child had taken part in an expanded newborn screening programme. (Table 1) Only 21% were aware that the remaining of the blood spot is potentially useful in other purposes unrelated to newborn screening.

**Table 1:** Characteristics of respondents and their knowledge on and experience of newborn screening and rDBS

	All (n = 1012)
**Characteristics**	
Mother	85%
Age (mean ± SD)	33.8 ± 6.0 years
Place of birth	
Hong Kong	69%
Mainland China	29%
Others	1.7%
Education attainment	
9th grade or below	6.8%
10th – 11th grade	26%
12th grade to diploma	22%
University degree or above	45%
Household income	
Less than HKD40000	52%
HKD40000- HKD79999	35%
HKD80000 or above	12%
Religion	
Christianity/Catholic	21%
Others (including Muslim, Buddhism, Traditional)	11%
No Religion	68%
Number of children	
1	53%
2	38%
3 or more	9.0%
**Knowledge on newborn screening**	
• Heard of newborn screening for inborn errors of metabolism before taking part in the survey.	83%
• Knew that newborn screening tests are conducted in Hong Kong.	47%
• Knew that newborn screening usually took place during 1–7 days after birth.	48%
**Knowledge on dried blood spot (DBS)**	
• DBS is collected for newborn screening.	40%
• rDBS is potentially useful in purposes unrelated to newborn screening.	21%
**Experience of newborn screening**	
• Having a child participated in newborn screening.	76%

### Views from parents on secondary use of rDBS

Only 9% of the respondents thought rDBS should not be stored for potential secondary use. From those who supported, most (74%) of them thought that rDBS should only be stored for certain uses. Respondents mainly (above 80%) support secondary uses related to newborn screening, e.g. quality control (85%) and health-related research (75%), while relatively fewer supported the use of rDBS in tracing victims (69%) or suspects of crimes (52%). (Table 2)

### Views from parents on consent mechanism

The majority (90%) wanted to be asked for permission if their child’s rDBS would be used for purposes unrelated to newborn screening. Most respondents thought they preferred an opt-in approach (74%), with only 6% preferred an opt-out approach and 20% accepting either.

In a hypothetical situation that the consent for storage of newborn’s rDBS states that “*It is not able to predict how rDBS will be used at the moment*.” (i.e. broad consent), the respondents indicated that they were more willing to support for unspecified uses limited to medical/health related research only (76%), or uses that were approved by a governance body (63%) than anonymous use (49%) and unlimited (i.e. any unspecified) future uses (12%). (Table 3) However the majority of respondents (80%) still preferred to be informed and asked for consent in future before any use, if possible. About 70% preferred their child to make their own decision about storage of their rDBS when they grow up, i.e. re-consent.

**Table 2:** Parents’ support on different secondary use of their child’s rDBS

	Absolutely Support	Support	Neutral	Not support	Absolutely Not support
• To check the results of newborn screening	51%	34%	14%	1.0%	0.3%
• Quality control and development of the screening tests	45%	36%	18%	0.9%	0.4%
• Research on inborn diseases of metabolism	54%	33%	13%	0.3%	0.3%
• Medical/health research and related policy	43%	32%	22%	3%	1.1%
• To identify victims of fire or natural disasters	38%	30%	24%	4.4%	2.8%
• To help trace suspects of serious crimes	29%	22%	30%	8.9%	9.0%

**Table 3:** Parents’ view giving a broad consent for storage of their child’s rDBS if the consent states that “It is not able to predict how rDBS will be used at the moment. The rDBS will be stored for uses as follows…

	Absolutely Support	Support	Neutral	Not support	Absolutely Not support
• Unspecified future use	5.1%	6.7%	22%	26%	40%
• Medical/Health related research only	28%	48%	19%	2.4%	2.5%
• Anonymous use only	17%	32%	30%	12%	9.1%
• Use that is approved by a governance body	27%	36%	25%	5.0%	6.4%
• Use that further consented by you i.e. you will be informed and asked for consent in future before any use	53%	27%	17%	1.2%	1.7%

### Factors associated with strong support in biobanking of rDBS for secondary use in medical research

In the fully adjusted models, fathers had stronger support to biobanking of rDBS than mothers. (Table 4) Education attainment overall was not associated with support to biobanking of using rDBS in medical research. But there was an interaction between sex and education (p-for-interaction = 0.03), such that the positive association between education and support to biobanking was observed in fathers but not mothers, and fathers with higher education showed the greatest support to biobanking of rDBS. (data not shown)

Non-local born parents (mainly mainland-China born), compared to local born parents, showed stronger support in biobanking of rDBS. (Table 4) None of the other demographic and socio-economic factors, including respondents’ age, number of children, income and religion was independently associated with support to biobanking rDBS for secondary uses.

**Table 4:** Factors associated with fully support^#^ to biobanking of rDBS for medical secondary use

	Fully support^#^ to biobanking of rDBS		RR^ for strong support to biobanking of rDBS (95%CI)
	Yes N = 214	No N = 787	
Mother	83%	86%	0.68* (0.49, 0.94)
Age (mean ± SD)	33.7 ± 4.8y	33.9 ± 6.2y	0.99 (0.97, 1.02)
Non-Hong Kong born	44%	27%	1.73* (1.33, 2.24)
University degree or above	41%	46%	0.94 (0.72, 1.25)
Household income of HKD80000 or above	12%	12%	1.03 (0.68, 1.56)
No Religion	66%	69%	0.79 (0.61, 1.02)
Only one child	47%	55%	0.80 (0.62, 1.03)

^**#**^Fully support from parents when being asked on their thoughts about having their child’s rDBS being stored in a national biobank (n = 214, 21%) compared to the rest (n = 787) consisting of 52% supported but indicated some concerns, 21% inclined to reject and 6% strongly opposed the idea of biobanking

^RR (relative risk) > 1.00 indicates the factor is associated with a greater support to biobanking of rDBS; RR < 1.00 indicates lower support

*p < 0.05; ^+^0.05 < p < 0.1

## Discussion

### Summary of findings

Researchers in Hong Kong and elsewhere see the great potential and value in biobanking and the application of population-based rDBS biobanks in research have started in other countries including Denmark and the United Kingdom and is foreseeable in Hong Kong. Based on our findings from both the focus groups and survey, the use of rDBS in medical research is well accepted by Hong Kong parents. However the focus group discussion also revealed some potential risks of biobanking rDBS that were perceived by parents, including leaking of data and mis-use of samples. Parents who took part in the focus groups and the survey consistently expressed their wish to take control and to be informed and/or asked for permission before rDBS of their children are stored. However the existing legal and policy frameworks in Hong Kong for personal data protection and research ethics are founded on the concept of informed consent, which is costly and unpractical to long-term retrospective accesses to large population-based biobanks [18]. The consenting challenges and parents’ views together indicate that while long-term retention and secondary use of rDBS is feasible in Hong Kong, a new legal and ethical policy framework with a transparent governance mechanism tailored for Hong Kong people is necessary to cope with the evolving need for, and ensure public trust in, biobanking of rDBS and other biological samples in Hong Kong.

### Limitations

We used a mixed method approach to understand the parents’ opinions on secondary uses of rDBS. However there are limitations of the study. The study was carried out amid a period of social unrest in Hong Kong. The period of social unrest in Hong Kong lasted for several months in 2019 till early 2020. A series of mass protests and demonstrations took place in response to proposed legislative changes, which escalated to territory-wide violent confrontations between protestors and the police. The unrest was associated with major mental health problems [19] as well as a general mis-trust of the government and police amongst diverse segments of society [20] in Hong Kong. The trust from the public in the government, particularly to the police, was low, especially amongst the younger segments of the population. Privacy has been a huge concern during this period of time, as indicated by the destruction of smart light posts suspected of conducting surveillance, and some concerns about the release of patients’ data by the Hospital Authority to the police. Our findings are likely to reflect an over-estimation on the distrust in the government and related authorities and the perceived harm of the mis-use of personal information in rDBS. We had more mothers compared to fathers participated in this study and thus our findings reflect predominantly the views from mothers. However since fathers, in particularly those with higher education, were more supportive to biobanking of rDBS, we may overestimate the concerns from parents on secondary use of rDBS. These factors may make the policy implications on retention and secondary use of rDBS more conservative and potentially more acceptable to most Hong Kong parents.

### Support and concerns in secondary use of rDBS

Our results indicated that secondary use of rDBS in medical research is well accepted by large proportion of Hong Kong parents. Fathers in Hong Kong, particularly higher educated fathers seemed to have the greatest support, although this requires further studies with larger sample size to confirm. As consistently shown in other similar studies, the focus group discussions revealed that the support from Hong Kong parents in medical research is linked with a perceived benefit to others [6, 13, 21] or perceived responsibility to contribute to such research [22]. From the survey findings, Hong Kong parents also seemed to show support for the use of rDBS in medical research unspecified in a broad consent, echoing with their trust of the academic bodies in handling rDBS. However implementation of such broad consent requires further public discussion and testing.

From the focus group discussion, we also observed common concerns from parents on potential risks of donating rDBS including leaking of data and mis-use of genetic information. Despite storing anonymous rDBS may reduce the risk of data leaking, parents in Hong Kong do wish to have their children’s samples identifiable in biobank for their perceived benefits, as parents in the US [6, 14]. Although research with unlinked rDBS, i.e. deidentified samples, to protect data privacy has been once suggested, [8] storing anonymous rDBS may be contradicts with parents’ will. On the other hand, such expectation from parents emphasises the need for a transparent policy on disclosure of individual research findings from rDBS.

### Governance and consenting

Another universal theme that emerged from parents from different settings, including Hong Kong, is the positive link between perceived risks/harm in storing rDBS and the lack of trust in the authorities. A transparent system and policy for retention and use of rDBS and involving parents in such system were coherently demanded not only by parents in Hong Kong and elsewhere [6, 13], but also newborn screening advisory committees [23]. An accountable and transparent oversight governance body is crucial to build trust in the biobank and newborn screening. The high level of trust in academics among Hong Kong parents observed in both focus group discussion and survey indicated that involvement of academic organisation in biobank governance is probably beneficial. The survey additionally revealed that a large proportion of parents accepted the broad consent approach for unspecified medical/health related research use of rDBS but not for any unspecified use. This implied that obtaining broad consent from parents on the retention and secondary use of rDBS is maybe a feasible consenting approach but they also perceived some threats and risks of uncontrolled/unregulated unspecified future uses. A robust, transparent and accountable governance framework is of particular importance when a broad consent is used, to prevent unauthorised use of rDBS, ensure personal information is handled safely and protect the right to withdraw consent for both parents and their adult children.

### The opt-out approach

Our survey revealed that only a small portion (6%) of Hong Kong parents preferred an opt-out approach with the majority wanted to take control and to be informed and/or be asked for permission before rDBS of their children are stored, as parents from the US [6, 17] and in Beijing, China [24]. They would also like their children to be able to re-consent when they grow up. An active “opt-in” consenting process is preferred by Hong Kong parents, similarly to parents in the US [6, 14]. It contradicts with a recent qualitative study which reported that opt-out consent was acceptable to some British parents enrolling their neonates in a randomised controlled trial [25]. However it has been shown parents of children with a serious health condition had higher levels of support than the general public towards the use of rDBS for research [15]. Since opt-out consent approach fundamentally conflicts with some legal and ethical norms, it may only be suitable among such parents and in settings with high level of trust of parents in the governance institution [21] or with sufficient explanation on the concept of opt-out consent to the parents [22]. Meanwhile, dynamic consent, that enables parents make granular decisions about their ongoing participation in the biobank, through an interactive digital interface, is made possible with advance in technology [26] and societal shifts in the use of digital tools [27]. The public’s acceptance of the dynamic consent, require further studies to elucidate.

### More education is needed

From both the opinion survey and the focus groups, we observed that many parents were not aware of the potential value of rDBS before taking part in the study. A low awareness of the retention of rDBS and its potential value in population health have been similarly reported in previous studies [28, 29]. A discussion on the topic has been shown to help parents feel positive about retention and secondary use of rDBS [14]. More importantly public education on both the newborn screening using DBS itself [30] and the potential risks and benefits of secondary use of rDBS to the population allows informed choice about not only storing rDBS, but also the consenting process. A continue open dialogue between the public and the screening community would be beneficial [31] when implementing policy related to rDBS. In the process, it is important to make sure technical terms, e.g. OMICS technologies, genome wide association studies or GWAS and DNA methylation, and the impact of these new technologies on data privacy are understood in diverse populations with different backgrounds and education levels [32].

## Conclusion

We observed that long-term storage and secondary use of rDBS from newborn screening for biomedical research is generally acceptable to Hong Kong parents given their autonomy is respected and their privacy is protected. A transparent broad consent for biobanking of rDBS with an accountable governance and transparent access policy are necessary to balance the concerns of parents and the research opportunities offered by rDBS. The bioethical issues of biobanking of rDBS are complex. Further studies on the governance framework for long-term biobanking rDBS, together with education to and continuous communications and collaboration with the public[33] will be required to build public trust in newborn screening programmes and future rDBS biobanks.

## Data Availability

The datasets used and analysed during the current study are available from the corresponding author on reasonable request.
